# Programmed death-ligand 1 signaling and expression are reversible by lycopene via PI3K/AKT and Raf/MEK/ERK pathways in tongue squamous cell carcinoma

**DOI:** 10.1186/s12263-022-00705-y

**Published:** 2022-02-14

**Authors:** Mingjing Peng, Songqing Fan, Junjun Li, Xiao Zhou, Qianjin Liao, Faqing Tang, Wei Liu

**Affiliations:** 1 Central South University, Xiangya Medical School affiliated Cancer Hospital, Hunan Cancer Hospital, Changsha, 410073 Hunan People’s Republic of China; 2 Institute of Clinical Pharmacology, Hunan Key Laboratory of Pharmacogenetics, Central South University, Changsha, 410078 Hunan People’s Republic of China; 3 Department of Pathology, Second Xiangya Hospital, Central South University, Changsha, 410011 Hunan People’s Republic of China; 4 Hunan Cancer Hospital, The Affiliated Cancer Hospital of Xiangya School of Medicine, Central South University, 410073 Changsha, People’s Republic of China; 5 Post-doctorate of Pathology,Second Xiangya Hospital, Central South University, Hunan Changsha, People’s Republic of China

**Keywords:** PD-L1, Lycopene, Proliferation, Apoptosis, EMT, TSCC

## Abstract

**Background:**

Cancer therapy targeting programmed death receptor-1 (PD-1 or CD279) or programmed death-ligand 1 (PD-L1 or CD274) gives hope to Tongue Squamous Cell Carcinoma (TSCC) treatment. However, the tumor-intrinsic mechanism of PD-L1 is not fully elucidated in TSCC. On the other hand, lycopene showed antitumor effects and chemotherapy/radiotherapy-enhancing effects by mechanisms closely correlated with PD-L1.

**Purpose:**

We aimed to explore whether the mechanisms of PD-L1 signaling and regulation are reversible by lycopene treatment in TSCC.

**Methods:**

We collected TSCC tissues and normal tissues for assessment of PD-L1 expression by immunohistochemical technique and western blotting. We measured the expression of PD-L1 in three TSCC cell lines and constructed cell lines with knockdown and overexpression of PD-L1. Then, we measured the proliferation by CCK-8 assay, migration and invasion by Transwell assay, and apoptosis by TUNEL assay in five groups with treatment of blank control, negative control with vector transfection, PD-L1 knockdown/overexpression, 4 μM lycopene, and combined 4 μM lycopene and PD-L1 knockdown/overexpression. We also systematically analyzed the PD-L1 constitutive signaling pathways and their effect EMT pathways. In order to bring out the mechanism underlying PI3K/AKT depressing Raf/MEK/ERK, we used PI3K inhibitor LY294002.

**Results:**

We detected significant PD-L1 upregulation in biopsies by western blot and immunohistochemistry. Our study demonstrated that PD-L1 upregulation elevated IGF-1R to activate the PI3K/AKT pathway but inactivated the Raf/MEK/ERK pathway in TSCC cell line CAL27, while PD-L1 knockdown decreased IGF-1R to inactivate both PI3K/AKT and Raf/MEK/ERK pathways in cell line SCC9, to increase/decrease p-FOXOs and decrease/increase p-GSK-3β, producing further changes in EMT, proliferation, migration, invasion, and apoptosis. Lycopene reversed PD-L1 signaling and expression by mechanisms opposite to PD-L1 upregulation but similar to PD-L1 knockdown.

**Conclusion:**

Taken together, this study firstly confirmed PD-L1 expression and signaling are reversible by lycopene via PI3K/AKT and Raf/MEK/ERK pathways in TSCC. Our study provides a sounder basis for comprehending PD-L1 signaling and expression and prevention and treatment of TSCC.

## Introduction

Tongue squamous cell carcinoma (TSCC) rates the most common type of oral cancer, accounting up to 38% of head and neck tumors and 90% of all oral cancers [1]. Several factors such as cigarette smoking, betel nut chewing, alcohol, human papillomavirus infection, and poor oral hygiene are involved in the pathogenesis of TSCC [2]. Surgical resection is the standard treatment for early TSCC, whereas chemoradiotherapy or chemotherapy with/without surgery are adopted for advanced cases. Cervical lymph node metastasis and recurrence and resistance to chemotherapy are the main obstacles to treating advanced TSCC [3]. Immune therapy has been recommended as optional treatment for head and neck SCC, but the majority of patients do not respond to PD-1/PD-L1 antibodies, and durable responses are observed only in a small proportion of patients. Therefore, it is necessary to find new treatment and explore the mechanism of carcinogenesis of TSCC.

Programmed death-ligand 1 (PD-L1 or CD274) is a protein expressed on the cell membrane of cancer cells that can bind with programmed death receptor 1 (PD-1 or CD279) expressed by infiltrated T cells [4]. When PD-L1 interacts with PD-1, the cytoplasmic domain of PD-1 can be phosphorylated and inhibit the signaling downstream of the T-cell receptors (TCR) [5]. After inhibition of the TCR-mediated signaling pathway, PD-1 prevents activating the pathways mediated by the PI3K/AKT or Ras/MEK/ERK cascade. This further inhibits the function of CD8^+^ T cells and leads to the promotion of tumor growth and metastasis [5]. The nonimmune PD-L1 signaling in cancer cells has not been fully clarified, but it is established that it promotes epithelial–mesenchymal transition (EMT), boosting tumor growth and metastasis [6]. A recent study confirmed that PD-L1 tends to be overexpressed at the early stage of TSCC, which is more likely to develop lymph node metastasis, indicating a close involvement with the initial development of tongue cancer [7].

Studies on mechanisms of PD-L1 threw more and more lights on its tumor-intrinsic signaling in cancer initiation, development, and treatment beyond immune suppression of T cells [8]. In breast cancer cells, PD-L1 binding with PD-1 induced phosphorylation of AKT and ERK, activating PI3K/AKT and MAPK/ERK pathways, and increased expression of chemotherapy refractory molecule MDR1 [9]. In melanoma cells, nicotine increased α9 nicotinic acetylcholine receptor activity and activated AKT and ERK signaling pathways, and at the same time, upregulated PD-L1 expression via transcription factor STAT3 binding to the PD-L1 promoter [10]. In nasopharyngeal carcinoma cells, PD-L1 was demonstrated to induce EMT through activation of the PI3K/AKT pathway [11]. In esophageal cancer, PD-L1 expression was confirmed to correlate with EMT both at tumor invasive front and in tumor samples, and what is more, PD-L1 expression significantly promoted cell viability, migration and EMT phenotype in Eca-109 cells. Furthermore, the same research also indicated that PD-1 fusion protein-mediated stimulation of PD-L1 and its cytoplasmic domain played a crucial role in promoting EMT phenotype [12, 13]. Interestingly, in glioblastoma multiforme, overexpressed PD-L1 promoted EMT and invasion via RAS/ERK/EMT activation [14].

Lycopene is a kind of carotene abundant in mature red fruits and vegetables, especially in tomatoes, carrots, watermelon, papaya, and guava [1]. Its central symmetric hydrocarbon chain contains 2 non-conjugated and 11 conjugated bonds. The powerful antioxidant property of lycopene is attributed to 11 conjugated double bonds [15]. Elevated production of reactive oxygen species (ROS) and reactive nitrogen species (RNS) cause oxidative stress and can oxidize cellular biomolecules, including lipids, carbohydrates, proteins, and DNA. The damages to these vital molecules can facilitate carcinogenesis by means of genetic alterations. Lycopene vigorously clears out oxidative stress and prevents carcinogenesis [16]. Increasing evidence suggested that lycopene plays an important role in cancer prevention and has the potential as an adjuvant to cancer therapy in different cancers such as lung, breast, pancreas, ovaries, prostrate, and uterine cervix [16]. Research results also inferred that lycopene might prevent the carcinogenic process through the inactivation of the growth factor (PDGF, VEGF, and IGF) induced the PI3K/AKT/mTOR and Ras/Raf/MAPK signaling pathways. The two constitutive signaling pathways as well as all transcript factors including Myc, STAT, NF-κB, IRF-1, AP-1, and HIF that were reported to contribute to modulation of PD-L1 expression at the transcription level tally marvelously with molecular targets of lycopene [16]. It was reported that lycopene treatment dose-dependently decreased the expression ratios of p-PI3K/PI3K, p-AKT/AKT, and p-m-TOR/m-TOR to suppress EMT process and increase the apoptosis of oral cancer cells [15]. So we hypothesize that lycopene can reverse PD-L1 overexpression-induced EMT by the same precise mechanisms and function as a compelling inhibitor for tumor-intrinsic PD-L1 signaling in TSCC.

Other things being equal, we elevated PD-L1 expression and tested its nonimmune mechanisms presumably via IGF-1R–activating PI3K/AKT and Raf/MEK/ERK pathways, which then elevated Myc, STAT3, NF-κB, and EMT transcript factors’ (Snail, Slug, and ZEB-1) activity, finally changing EMT markers E-cadhrin, N-Cadhrin, and Vimentin in TSCC cells. Then we added lycopene treatment to downregulate PD-L1 expression and observed the subsequent changes of its downstream molecules and effects. We also test the combination treatment of PD-L1 elevation and lycopene treatment. Subsequently, we knocked down PD-L1 expression in tongue squamous cells to test the molecular changes in proposed pathways; we also observed the independent effects of lycopene treatments and its combination effects with PD-L1 knockdown. These are quite arduous and complicated tasks. We assume the responsibilities to pave the main foundation for future improvements.

## Material and methods

### Tissue sample collection

Seventeen patients who had been diagnosed before or during tongue resection as TSCC without metastasis were enrolled in our study. We carefully examined each cancer lesion whether it was larger than 0.5 × 0.5 × 1 cm^3^, so we made sure that there were enough tissues after sample collection for normal pathological diagnosis. We collected a lump of TSCC tissues about the size of 0.2 × 0.2 × 0.5 cm^3^ and a similar lump of normal tissues from each patient’s same sample, eventually gaining 34 lumps. This work was completed between November of 2020 and February of 2021. Specimens were collected indiscriminately in the Pathology Department of Hunan Cancer Hospital from samples with tongue cancer lesion bigger than 0.5 × 0.5 × 1 cm^3^. All specimens were collected with the consent of the patient and approval of the Ethics Committee of our institute.

### Quantitative real-time PCR (qRT-PCR)

In brief, total RNA was extracted by the TRIzol method. cDNA Reverse Transcription Kit (#CW2569, ComWin Biotech, China) was used to reverse RNA into cDNA. The Ultra SYBR Mixture (#CW2601, ComWin Biotech, China) was used to test relative CD274 transcript levels in the ABI 7900 system. Relative CD274 transcript level was calculated using the 2^-ΔΔCt^ method with β-actin as the internal gene. The primer sequences used in this study were as follows: CD274-F: TTGCTGAACGCCCCATACAA, CD274-R: TCCAGATGACTTCGGCCTTG; β-actin-F: ACATCCGTAAAGACCTCTATGCC, β-actin-R: TACTCCTGCTTGCTGATCCAC.

### Cell culture and treatment

Because CAL27 and SCC9 had been used in previous research, they were chosen in our comparable research; SCC25 was added for new exploration. The human tongue cancer cell lines CAL27, SCC9, and SCC25 were purchased from the National Collection of Authenticated Cell Cultures (Shanghai, China). All tongue cancer cell lines were cultured in Dulbecco’s Modified Eagle Medium (DMEM, HonorGene, Changsha, China) supplemented with 10% FBS and 1% Penicillin–Streptomycin Solution (HonorGene, Changsha, China). Lycopene treatment (L812281, MACKLIN, Shanghai, China) was used at a final concentration of 4 μM and 25 μM for 48 h. LY294002 treatment (PI3K-AKT inhibitor, MedChemExpress LLC, Shanghai, China) was used at a final concentration of 20 μM for 24 h. Quantitative real-time PCR results showed that PD-L1 mRNA was expressed in CAL27, SCC25, and SCC9 cell lines at low, middle, and high levels. The preliminary experiment suggested that PD-L1 knockdown was very difficult because of the low expression level in CAL27 cells. So we chose CAL27 for the overexpression experiment and SCC9 for the knockdown experiment. SCC25 with an average PD-L1 expression level was chosen for future TSCC animal model experiments to be treated by different levels of lycopene. SCC9 cells were transfected for 48 h with small interfering RNA (SiRNA) targeting sequences si-CD274_002 (GATATTTGCTGTCTTTATA), si-CD274_007 (GCTGTCTTTATATTCATGA), and si-CD274_008 (GACAAGCAGTGACCATCAA). siRNA NC means SiRNA-negative control. The knockdown efficiency of si-CD274_002, si-CD274_007, and si-CD274_008 was 41.7%, 75.0%, and 37.5%, respectively. So si-CD274_007 was chosen for the following knockdown experiments. Then SCC9 cells (5 × 10^4^ cells) were divided into five groups to receive treatment for 48 h that was blank control (no treatment), NC (transfected with 100 μM siRNA NC), NC + Lycopene (100 μM siRNA NC + 4 μM lycopene), si-CD274 (100 μM), si-CD274 (100 μM) + Lycopene (4 μM). CAL27 cells were transfected with pHG-CMV-Kan2-CD274 plasmid, and the pHG-CMV-Kan2 plasmid was a negative control. CAL27 cells (5 × 10^4^ cells) were divided into five groups to receive treatment for 48 h that was blank control (no treatment), NC (transfected with 2.5 μg pHG-CMV-Kan2 plasmid), NC + Lycopene (transfected with 2.5 μg pHG-CMV-Kan2 plasmid + 4 μM lycopene), CD274 (2.5 μg pHG-CMV-Kan2-CD274 plasmid), and CD274 + Lycopene (2.5 μg pHG-CMV-Kan2-CD274 plasmid + 4 μM lycopene). Then we added PI3K-Akt inhibitor LY294002 in CAL27 cells to study the specific mechanism of PI3K/Akt inhibiting the Raf/MEK/ERK cascade. CAL27 cells (5 × 10^4^ cells) were divided into four groups to receive treatment that were NC (transfected with 2.5 μg pHG-CMV-Kan2 plasmid for 48 h), CD274 (2.5 μg pHG-CMV-Kan2-CD274 plasmid for 48 h), CD274 + LY294002 (2.5 μg pHG-CMV-Kan2-CD274 plasmid for 24 h, then we added 20 μM LY294002 for another 24 h), and CD274 + Lycopene (2.5 μg pHG-CMV-Kan2-CD274 plasmid and 4 μM lycopene for 48 h). Finally, we compared the effects of the 25-μM lycopene treatment and PD-L1 knockdown as well as the effect of their combination. SCC25 cells were divided into four groups to receive treatment that were NC (transfected with 100 μM siRNA NC for 48 h), NC (100 μM siRNA NC for 48 h) + Lycopene (25 μM for 48 h), si-CD274 (100 μM for 48 h), si-CD274 (100 μM) + Lycopene (25 μM) for 48 h.

### Western blot

According to the instructions, RIPA lysis buffer (#P0013B, Beyotime) was used to extract the total protein. The protein of each group was quantified according to the BCA protein determination kit. Total protein was separated on SDS-PAGE gels and transferred to PVDF, which was sealed with 5% skim milk solution for 2 h and incubated with primary antibodies against PD-L1 and downstream key molecules p-IGF-1R, IGF-1R, p-Akt1 (Ser473), p-Akt1 (Thr308), AKT1, p-c-Raf, p-MEK1, MEK1, p-ERK, ERK, p-FOXO1A, FOXO1A, p-FOXO3A, FOXO3A, p-GSK-3β (Ser9), and GSK-3β; important transcript factors NF-κBp65, STAT3, and c-Myc; EMT-transcription factors Slug, Snail, and ZEB-1; and EMT markers E-cadherin, N-cadherin, and Vimentin. Primary antibodies against p-IGF-1R (3021T), IGF-1R (9750S), Akt1 (2938S), FOXO3A (12829S), ERK1/2 (4695S), Slug (9585T), Snail (3879T), p-GSK-3β (#5558), GSK-3β (12456S), and p-c-Raf (#9427) were purchased from Cell Signaling Technology (MA, USA); p-AKT1 (Ser473) (ab81283), p-AKT1 (Thr308) (ab38449), Vimentin (ab92547), N-cadherin (ab76011), ZEB-1 (ab203829), NF-κBp65 (ab16502), p-FOXO3A (ab47285), p-FOXO1A (ab131339), FOXO1A (ab179450), p-MEK1 (ab96379), MEK1 (ab32091), and p-ERK (ab32538) were purchased from Abcam (Cambridge, UK); PD-L1 (17952-1-AP), STAT3 (10253-2-AP), c-Myc (10828-1-AP), E-cadherin (20874-1-AP), and β-actin (66009-1-Ig) were purchased from Proteintech (LA, USA). All the primary antibodies were incubated at 4 °C overnight, followed by incubation with corresponding secondary antibody HRP Goat anti-mouse IgG (SA00001-1, Proteintech) or HRP Goat anti-rabbit IgG (SA00001-2, Proteintech). After ECL exposure, the Odyssey Infrared Imaging System (LI-COR Biosciences, Lincoln, NE, USA) was used to detect the protein band, and β-actin was adopted as the internal reference to detect the protein expression.

### HE staining

The histopathological changes of TSCC were examined by HE. The slices were baked at 60 °C for 1-2 h and dewaxed into water. Then the slices were placed in 100%, 100%, 95%, 85%, and 75% ethanol for 5 min. Then the slices were immersed in distilled water for 5 min, stained with hematoxylin for 5-8 min, rinsed with distilled water and returned blue with phosphate-buffered saline (PBS). The slices were stained with eosin for 30 s and rinsed with distilled water. The slices were dehydrated by gradient alcohol (95-100%), each grade 5 min. Then they were put in xylene for 10 min, twice, sealed with neutral gum, and observed under the microscope.

### Immunohistochemistry (IHC)

The expression of PD-L1 in TSCC and normal tissues was detected by IHC. The slices were roasted at 60 °C for 1 h. The slices were dewaxed into the water, and heated to repair the antigen. One percent of periodic acid was added at room temperature for 10 min to inactivate endogenous enzymes. Appropriate dilution of primary antibody PD-L1 was added to incubate at 4 °C overnight. The secondary antibody was added to incubate at 37 °C for 30 min. DAB was used for color development, and hematoxylin was redyed for 5-10 min. Then the slice was washed with distilled water, and PBS was used to return blue. The slices were dehydrated by graded levels of alcohol (60-100%) for 5 min per grade. Then they were placed in xylene for 10 min, twice, sealed with neutral gum, and examined under the microscope.

### Cell counting kit-8 (CCK-8) assay

The cells in different groups were digested and counted and inoculated in 96-well plates at the density of 5 × 10^3^ cells/well, 100 μL per well. Each group was set with 3 multiple holes. Ten microliters of CCK-8 (#NU679, Dojindo, Japan) was added to each well to incubate for the appropriate time (24 h, 48 h, 72 h). The absorbance (OD) at 450 nm was analyzed by BioTek microplate (MB-530, Heales, China) after further incubation at 37 °C 5% CO_2_ for 4 h.

### Transwell assay

The migration and invasion abilities of five groups of CAL27 and SCC9 cells with aforesaid treatments were measured by Transwell assay. SCC25 was left for future in vivo experiments. The cells were harvested and resuspended in a serum-free medium. The cells were seeded into the upper chamber of a Transwell insert (Corning, USA) for the migration assay or a Transwell insert coated with Matrigel (BD, USA) for the invasion assay. Medium containing 10% FBS was added to the bottom chamber. After incubation for 24 h at 37 °C, the cells on the upper surface of the membrane were removed, the invaded or migrated cells on the lower surface of the membrane were fixed with 4% polyformaldehyde and then stained with 0.1% crystal violet. The number of invaded or migrated cells was counted under a light microscope.

### TUNEL

The apoptotic cell deaths in the aforementioned five groups of CAL27 and SCC9 cells were examined by TUNEL assay. SCC25 was left for future in vivo experiments. Cells were given five kinds of treatments for 48 h before the assay. Then cells were harvested to measure apoptosis using a TUNEL cell apoptosis in situ detection kit (Yeasen Biotechnology Shanghai, China) following the manufacturer’s instructions. Three independent experiments were performed, and average numbers of positively stained cells in three randomly chosen areas were presented in quantitation.

### Statistical analysis

The data of above tests and experiments were statistically analyzed with Graphpad Prism 8.0 and Statistical Product and Service Solutions 22.0 statistical software. The measurement data were expressed as mean ± standard deviation (Mean ± SD) and repeated at least 3 times. One-way analysis of variance (ANOVA) was used to compare multiple groups, and Student’s t test was used to compare between two groups. *P* < 0.05 indicated that the difference was statistically significant.

## Results

### PD-L1 expression in the TSCC samples and cell lines

**Fig. 1:**
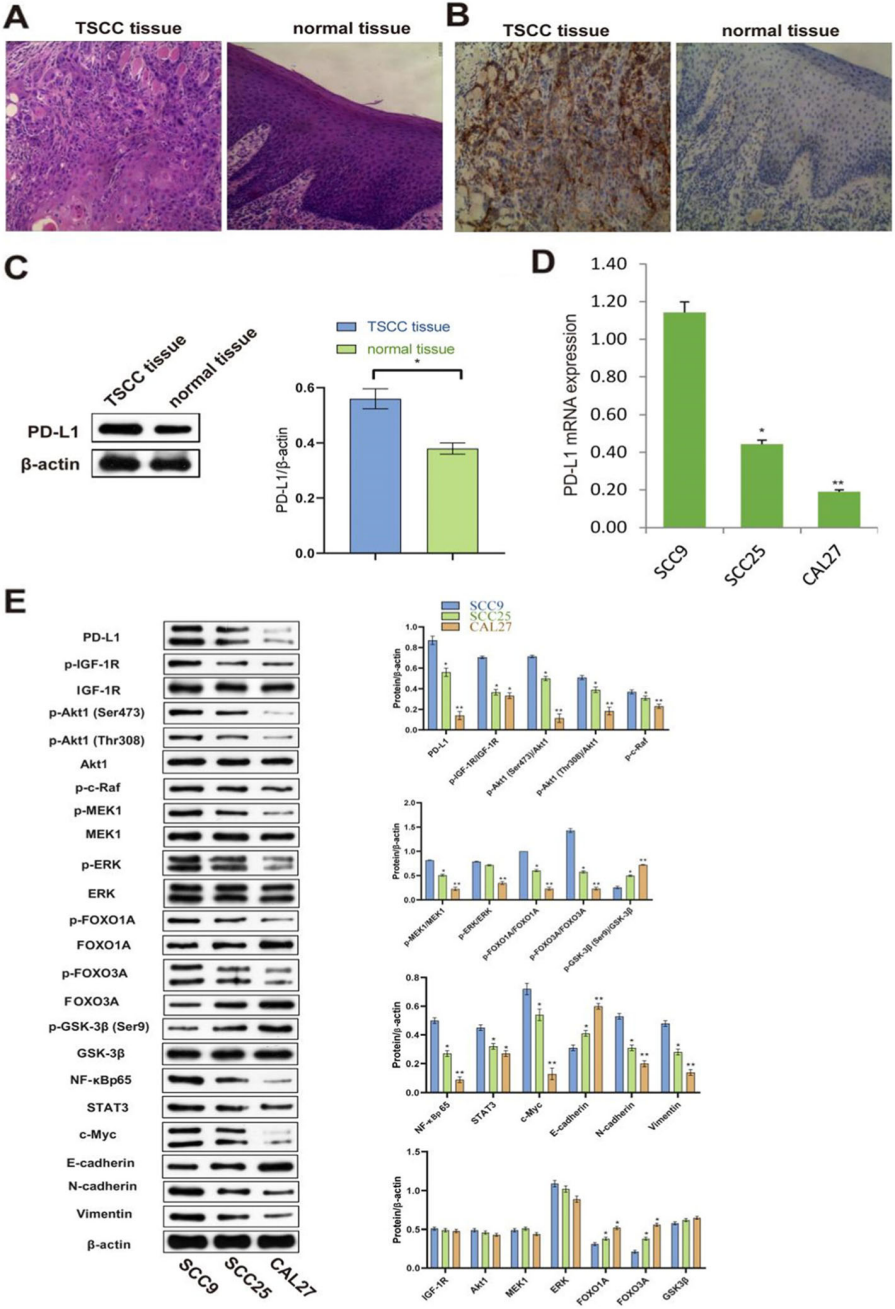
PD-L1 expression in the TSCC samples and cell lines. **A** HE staining was used to evaluate TSCC pathology. All cancerous tissues were pathologically confirmed as TSCC. **B** The expression of PD-L1 was evaluated by IHC. The expression of PD-L1 was significantly upregulated in TSCC. **C** The expression of PD-L1 in TSCC versus normal tissue were measured by western blot. The former was significantly upregulated. **D** PD-L1 mRNA expression in SCC9, SCC25, and CAL27 cells was detected by qRT-PCR. **E** To elucidate the mechanisms of PD-L1 nonimmune signaling in SCC9, SCC25, and CAL27 cells, PD-L1 and downstream key molecules were systematically analyzed by western bolt. **P < 0.01, *P < 0.05

Firstly, HE staining was used to evaluate histopathology of 17 paired TSCC and normal tissues of 17 patients. As shown in Fig. 1A, compared with normal tissue, TSCC tissue showed squamous characteristics of keratinized beads and intercellular bridges and increased nuclear heteromorphism. Tumor cells showed crab foot-like invasive growth with no boundary to normal tissue. There are high degrees of lymphocyte infiltration in the tumor stroma which might expose to inhibitory effects of PD-L1 overexpressed on cancer cell membranes. The expression of PD-L1 was evaluated by IHC. Compared with normal tissue, PD-L1 expression was significantly increased in the TSCC group (Fig. 1B). Then we detected PD-L1 expression by western blot. Compared with normal tissue, PD-L1 expression was significantly increased in the TSCC group (Fig. 1C). Subsequently, we tested the PD-L1 mRNA expression in three TSCC cell lines and found that the PD-L1 mRNA expression was high in SCC9 cells, middle in SCC25 cells, and low in CAL27 cells; there were significant differences between groups (one-way ANOVA and post hoc Fisher's least significant difference test; *P* < 0.01) (Fig. 1D). In order to elucidate the mechanisms of PD-L1 nonimmune signaling in SCC9, SCC25, and CAL27 cells, PD-L1 and downstream key molecules p-IGF-1R, p-Akt1 (Ser473), p-Akt1 (Thr308), p-c-Raf, p-MEK1, p-ERK, p-FOXO1A, p-FOXO3A, and p-GSK-3β (Ser9), important transcript factors NF-κBp65, STAT3, and c-Myc; EMT-TFs Slug, Snail, and ZEB-1; and EMT markers E-cadherin, N-cadherin, and Vimentin were systematically analyzed by western bolt. In consistence with results of PD-L1 mRNA expression and our reasoning about PD-L1 signaling, except for p-GSK-3β (Ser9) and E-cadherin, all molecules were high in SCC9 cells, middle in SCC25 cells, and low in CAL27 cells. As for the total amount of IGF-1R, Akt1, MEK1, ERK, FOXO1A, FOXO3A, and GSK-3β in three cell lines, IGF-1R, Akt1, MEK, ERK, and GSK-3β remained stable; FOXO1A and FOXO3A increased, whereas p-FOXO1A and p-FOXO3A decreased; therefore, p-FOXO1A/FOXO3A and p-FOXO3A/FOXO3A decreased (Fig. 1E). As we intended to gain a dramatic effect and at the same time to reduce experimental difficulty, based on qRT-PCR and westblot results, we chose CAL27 for the overexpression experiment and the 4-μM lycopene treatment, SCC9 for the 4-μM lycopene treatment and the SiRNA knockdown experiment; SCC25 was chosen later for comparison of the 25-μM lycopene treatment and SiRNA knockdown on the downregulation of PD-L1 expression and signaling.

### Overexpression of PD-L1 promoted CAL27 cell proliferation, migration, and invasion and inhibited apoptosis, while lycopene reversed the effect

**Fig. 2:**
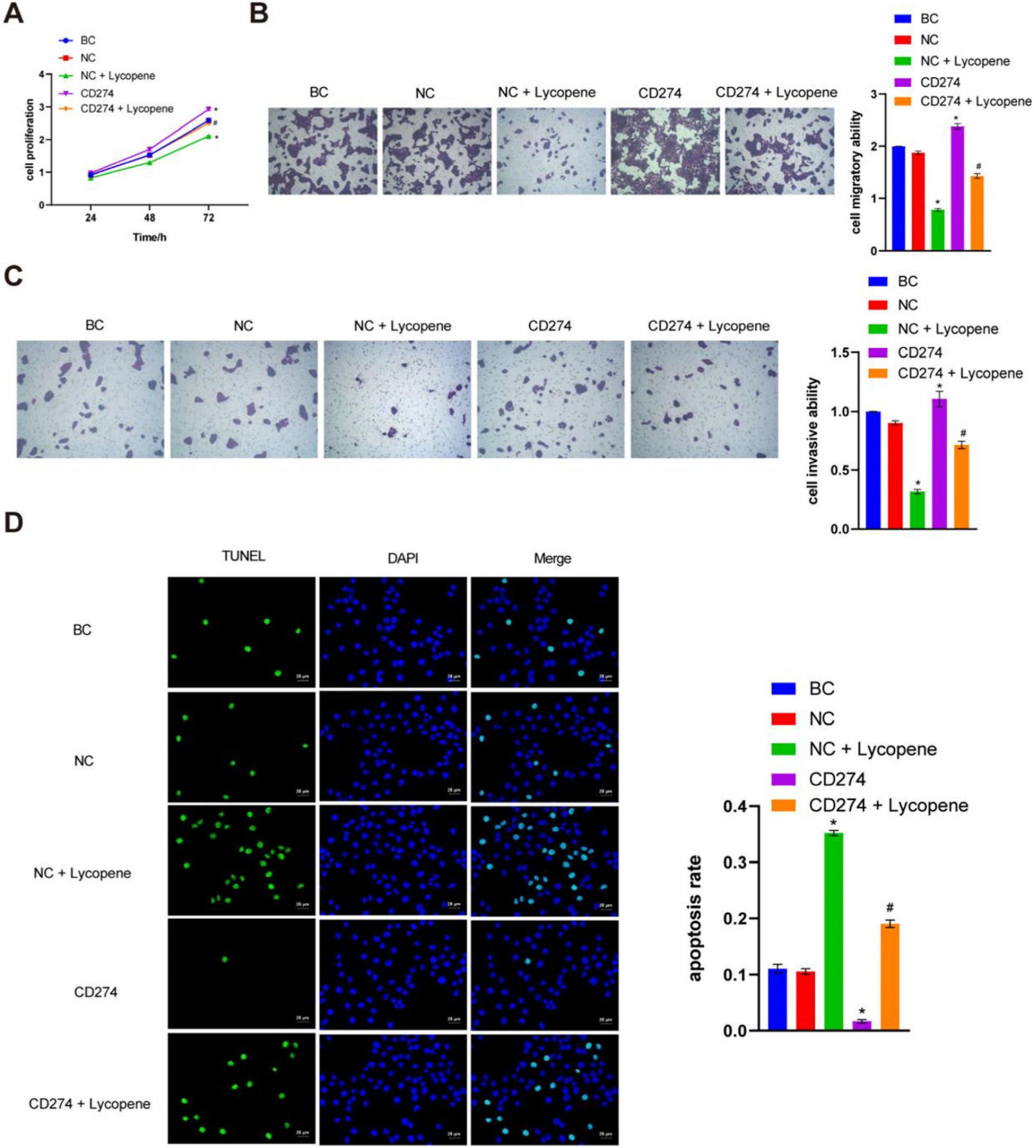
Overexpression of PD-L1 promoted CAL27 cell proliferation, migration, and invasion and inhibited apoptosis, while lycopene reversed the effect. **A** CCK-8 assay was used to measure CAL27 cell proliferation. **B** and **C** Transwell assay was carried out to measure CAL27 cells’ migratory and invasive ability. **D** TUNEL was used to detect CAL27 cell apoptosis. *P < 0.05 vs NC, #*P* < 0.05 vs CD274. NC means negative control, BC means blank control. CD274 also known as PD-L1

To study the effect of PD-L1, we overexpressed PD-L1 in CAL27 cells. The PD-L1 plasmid transfection efficiency was between 70 and 80%. There were no significant differences between blank control (BC) group and negative control (NC) group (Student’s t test, *P* > 0.05). Compared with the NC group, lycopene significantly inhibited CAL27 cell proliferation, migration, and invasion and promoted CAL27 cell apoptosis(one-way ANOVA and post hoc Fisher's least significant difference test, *P* < 0.05), while overexpression of PD-L1 significantly promoted CAL27 cell proliferation, migration, and invasion, inhibited CAL27 cell apoptosis (*P* < 0.05). Compared with the CD274 group, after adding lycopene, proliferation, migration, and invasion of CAL27 cells were reduced; CAL27 cell apoptosis was increased (*P* < 0.05) (Fig. 2A-D). These results revealed that overexpression of PD-L1 promoted CAL27 cell proliferation, migration, and invasion-inhibited apoptosis, which suggested that PD-L1 could be considered as an oncogene. Lycopene reversed the aforementioned carcinogenic effects of PD-L1 overexpression which suggested that lycopene could be considered as an inhibitor of PD-L1 signaling.

### Knockdown of PD-L1 inhibited SCC9 cell proliferation, migration, and invasion and promoted apoptosis, while lycopene aggravated the effect

**Fig. 3:**
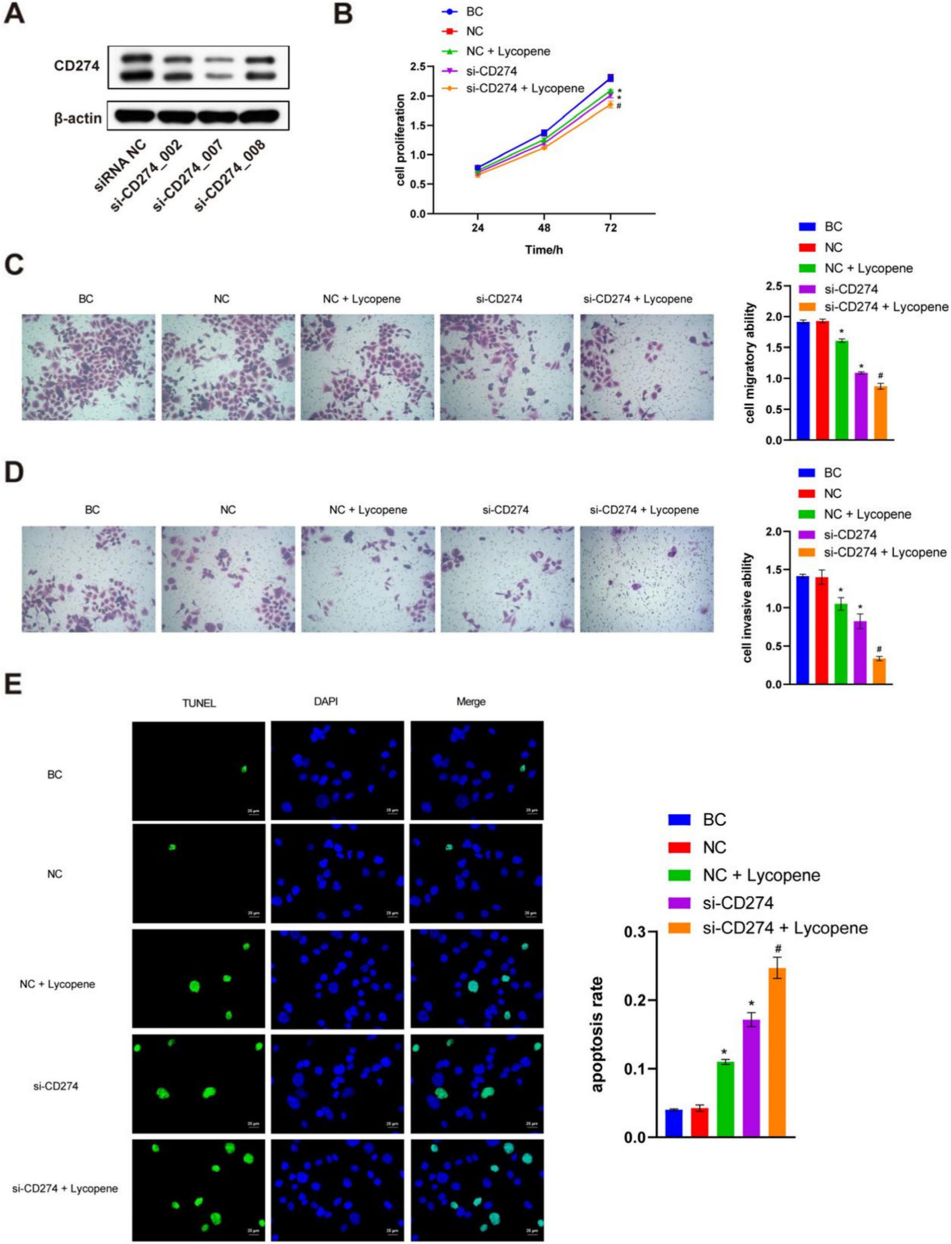
Knockdown of PD-L1 inhibited SCC9 cell proliferation, migration, and invasion and promoted apoptosis, while lycopene aggravated the effect. **A** The knockdown efficiency of si-CD274. There are two bands of PD-L1 up 65 ~ 70 KD, down 45 ~ 56 KD, according to instructions of the supplier, the down band is chosen to count knockdown efficiency at 75%. **B** CCK-8 assay was used to measure SCC9 cell proliferation. **C** and **D** Transwell assay was carried out to measure SCC9 cells migratory and invasive ability. **E** TUNEL was used to detect SCC9 cells apoptosis. *P < 0.05 vs NC, #*P* < 0.05 vs si-CD274. NC means negative control; BC means blank control. CD274 also known as PD-L1

To study the effect of PD-L1, we also knocked down PD-L1 in SCC9 cells. SCC-9 cells were transfected with small interfering RNA targeting sequences si-CD274_002, si-CD274_007, and si-CD274_008. As shown in Fig. 3A, the knockdown efficiency of si-CD274_007 was the highest, which is 75%. So si-CD274_007 was chosen for the following knockdown experiments. There were no significant differences between the blank control (BC) group and the negative control (NC) group (Student’s t test, *P* > 0.05). Compared with the NC group, lycopene inhibited SCC9 cell proliferation, migration, and invasion; promoted SCC9 cells apoptosis (one-way ANOVA and post hoc Fisher's least significant difference test, *P* < 0.05). In addition, knockdown of PD-L1 also inhibited SCC9 cell proliferation, migration, and invasion and promoted SCC9 cell apoptosis (*P* < 0.05). There were similar mechanisms and effects between the PD-L1 knockdown and lycopene treatment; therefore, we progressed next to study their combination effects. Compared with the si-CD274 group, SCC9 cell proliferation, migration, and invasion were decreased after adding lycopene; SCC9 cell apoptosis was increased (*P* < 0.05) (Fig. 3B-E). These results showed that knockdown of PD-L1 inhibited SCC9 cell proliferation, migration, and invasion and promoted apoptosis, while lycopene aggravated the effects.

### Effects of lycopene, CD274 overexpression, and their combination on PD-L1 constitutive signaling and EMT pathway

**Fig. 4:**
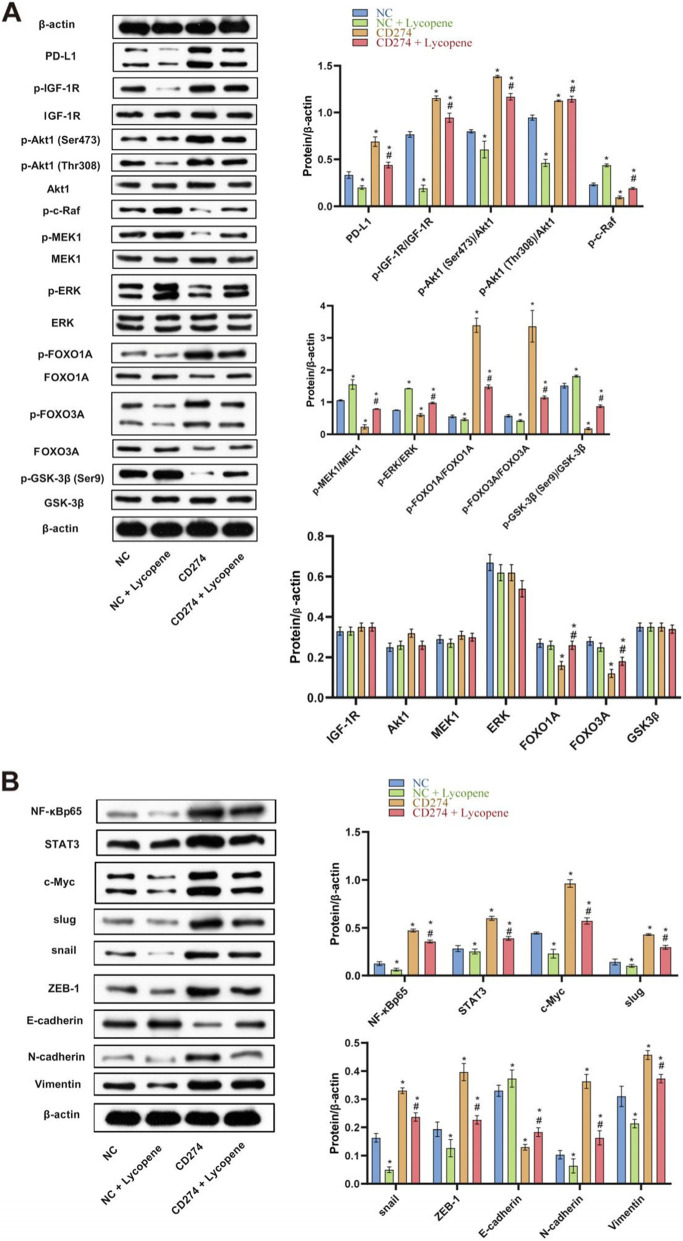
Effects of lycopene, CD274 overexpression and their combination on EMT pathway. **A** PD-L1 and downstream key molecules p-IGF-1R, p-Akt1 (Ser473), p-Akt1 (Thr308), p-c-Raf, p-MEK1, p-ERK, p-FOXO1A, p-FOXO3A, and p-GSK-3β (Ser9) expressions were analyzed by western bolt. According to manufacturer’s instructions, there were two bands of PD-L1, p-ERK and p-FOXO3A owing to post-translation modifications. **B** Western bolt was used to analyze important transcript factors NF-κBp65, STAT3, and c-Myc; EMT-transcription factors Slug, Snail, and ZEB-1; and EMT markers E-cadherin, N-cadherin, and Vimentin expressions. According to manufacturer’s instructions, there were two bands of c-Myc owing to different splicing. *P < 0.05 vs NC, #*P* < 0.05 vs CD274. NC means negative control. CD274 also known as PD-L1

To elucidate the mechanisms of PD-L1 nonimmune signaling in tongue cancer cells, PD-L1 and downstream key molecules p-IGF-1R, IGF-1R, p-Akt1 (Ser473), p-Akt1 (Thr308), AKT1, p-c-Raf, p-MEK1, MEK1, p-ERK, ERK, p-FOXO1A, FOXO1A, p-FOXO3A, FOXO3A, p-GSK-3β (Ser9), and GSK-3β; important transcript factors NF-κBp65, STAT3, and c-Myc; EMT-transcription factors Slug, Snail, and ZEB-1; and EMT markers E-cadherin, N-cadherin, and Vimentin were systematically analyzed by western bolt. As shown in Fig. 4A and B, PD-L1 protein expression increased after forced overexpression of PD-L1, then p-IGF-1R elevated; this implied the possible involvement of the IGF system in the mediation of PD-L1 signaling. Further signaling increased p-Akt1 (Ser473) and p-Akt1 (Thr308), but the level of p-c-Raf, p-MEK1, and p-ERK decreased, indicating activation of the PI3K/AKT pathway but inhibition of the Raf/MEK/ERK cascade. We should ask ourselves how to interpret the result. There were few reports stating that activated p-Akt could depress the Raf/MEK/ERK cascade in some prostate cancer cells [17]; whether this mechanism works out in our case can be tested by following experiment. Analysis continues, p-Akt1–dependent p-FOXO1A and p-FOXO3A also elevated. The effect on the critical EMT controlling molecule p-GSK-3β was a most dramatic decrease, which significantly promoted NF-κBp65, STAT3, and c-Myc expression, suggesting a typically negative regulation [18]. Downstream molecules EMT-TFs Slug, Snail, and ZEB-1 increased nearly twofold, reducing E-cadherin by almost two thirds, increasing N-cadherin by almost 3-fold, and elevating Vimentin expression, all of which indicated a typically successful EMT [17]. The opposite effects were observed after the lycopene treatment, both PD-L1 and p-IGF-1R expressions downregulated, the downstream intermediate molecules changed just opposite to PD-L1 upregulation, then finally significantly increasing E-cadherin and decreasing N-cadherin and Vimentin, all of which indicated a strengthened epithelial phenotype and make EMT more impossible. Compared with PD-L1 overexpression, adding lycopene significantly reduced PD-L1 and IGF-1R expression, decreased p-Akt1 (Ser473) and p-Akt1 (Thr308), and increased p-c-Raf, p-MEK1, and p-ERK; Akt1-dependent P-FOXO1A and p-FOXO3A also decreased; these results confirmed that lycopene reversed PD-L1 signaling probably by the same mechanism. The effect on critical EMT controlling molecule p-GSK-3β (Ser9) was a dramatic increase, then it notably reduced NF-κBp65, STAT3, and c-Myc and EMT-TFs Slug, Snail, and ZEB-1 activity as expected, finally increasing E-cadherin by one third, reducing N-cadherin by a half, and abating Vimentin expression, which indicated a typically successful MET. All aspects taken together, these results demonstrated that PD-L1 expression and signaling were actually reversible by the lycopene treatment by the same mechanism.

### Effects of lycopene, CD274 knockdown, and their combination on PD-L1 constitutive signaling and EMT pathway

**Fig. 5:**
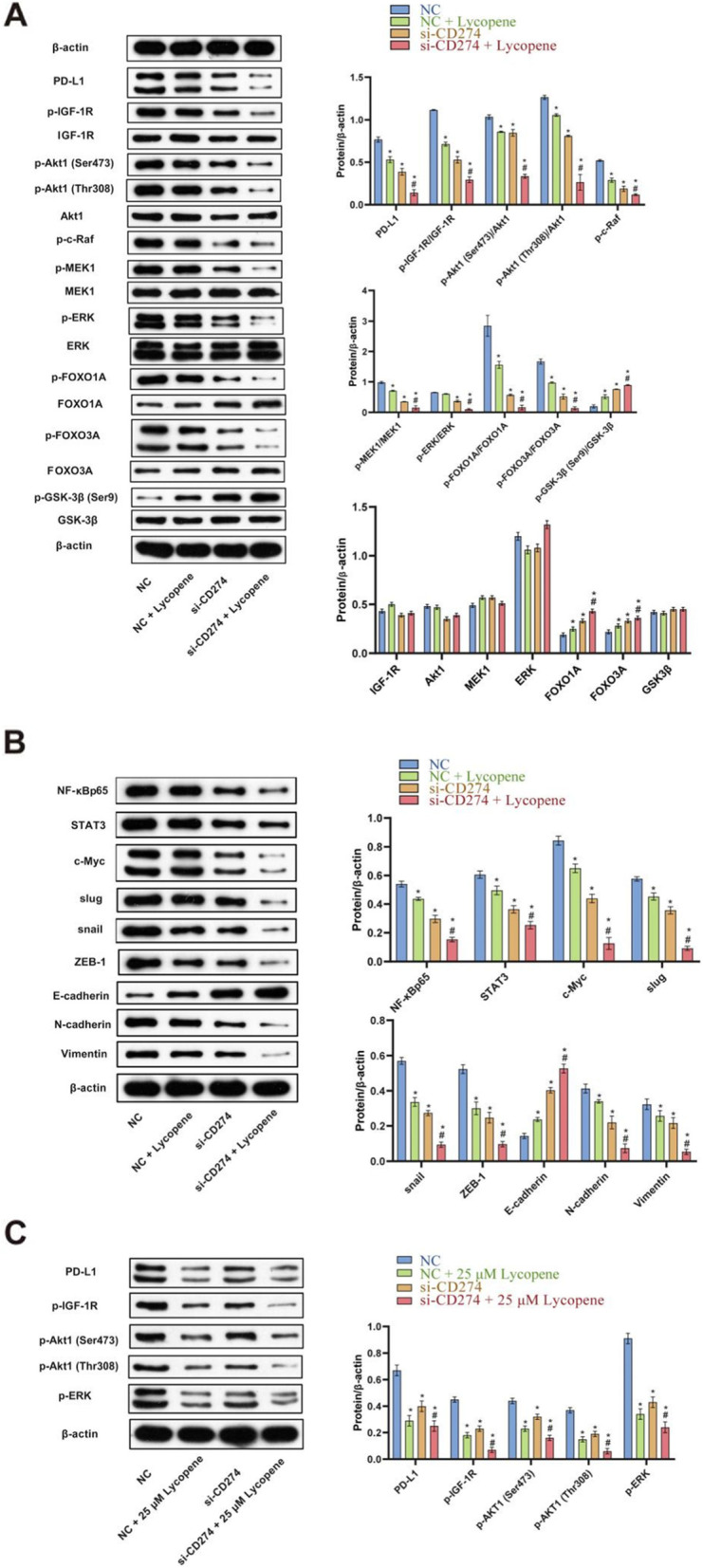
Effects of lycopene, CD274 knockdown, and their combination on EMT pathway. **A** Western bolt was used to detect PD-L1 and downstream key molecules p-IGF-1R, p-Akt1 (Ser473), p-Akt1 (Thr308), p-c-Raf, p-MEK1, p-ERK, p-FOXO1A, p-FOXO3A, and p-GSK-3β (Ser9) expressions. According to manufacturer’s instructions, there were two bands of PD-L1, p-ERK and p-FOXO3A owing to post-translation modifications. **B** Important transcript factors NF-κBp65, STAT3, and c-Myc; EMT-transcription factors Slug, Snail, and ZEB-1; and EMT markers E-cadherin, N-cadherin, and Vimentin expressions were measured by western bolt. According to manufacturer’s instructions, there were two bands of c-Myc owing to different splicing. **C** Western bolt was performed to analyze 25 μM lycopene and SiRNA knockdown on PD-L1 and downstream key molecules p-IGF-1R, p-Akt1 (Ser473), p-Akt1 (Thr308), and p-ERK expressions in SCC25 cells. According to manufacturer’s instructions, there were two bands of PD-L1, p-ERK owing to post-translation modification. *P < 0.05 vs NC, #*P* < 0.05 vs CD274. NC means negative control. CD274 also known as PD-L1

Then, we detected the expression of the abovementioned proteins after knockdown of PD-L1. As shown in Fig. 5A and B, PD-L1 protein expression decreased dramatically by knockdown of PD-L1, then IGF-1R expression fell, which further implied that the IGF system was involved in PD-L1 expression and signaling. Further signaling decreased p-Akt1 (Ser473) and p-Akt1 (Thr308); meantime, p-c-Raf, p-MEK1, and p-ERK fell, which indicated inactivation of both PI3K/AKT and Raf/MEK/ERK pathways. We deduced that PI3K/AKT did not depress the Raf/MEK/ERK cascade in SCC9 cells. P-FOXO1A and p-FOXO3A were also decreased since their levels depended on activated Akt1. The effect on the EMT negative control molecule p-GSK-3β was a dramatic increase, which significantly reduced NF-κBp65, STAT3, and c-Myc activity by nearly a half as expected. Then EMT-TFs Slug, Snail, and ZEB-1 decreased almost a half, increasing E-cadherin by threefold, reducing N-cadherin by a half, abating Vimentin expression, which indicated a typically successful MET. Lycopene at 4 μM produced similar but only moderate downregulating effects on PD-L1 expression and signaling, implying that a concentration of 16 μM or higher might be required to reduce it; this speculation was confirmed by the comparable effect of the 25-μM lycopene treatment to PD-L1 knockdown. As for the combination treatment, lycopene synergized PD-L1 knockdown effects on the whole pathway, increasing E-cadherin and reducing N-cadherin and Vimentin expression, all of which indicated a much more strengthened epithelial phenotype.

Subsequently, we proceeded to test the effects of the 25-μM lycopene treatment and PD-L1 knockdown on PD-L1 expression and signaling in SCC25 cells. As was demonstrated in Fig. 5E, the 25-μM lycopene treatment reduced PD-L1 expression and signaling more dramatically than PD-L1 knockdown; p-IGF-1R, p-Akt1 (Ser473), p-Akt1 (Thr308), p-c-Raf, p-MEK1, and p-ERK levels decreased to lower levels in the 25-μM lycopene treatment group than the PD-L1 knockdown group; combination of the 25-μM lycopene treatment and PD-L1 knockdown enhanced 25 μM lycopene downregulating effects on PD-L1 expression and signaling only in a small degree (Fig. 5E).

### Effects of CD274 overexpression, LY294002, and lycopene on PI3K/AKT and Raf/MEK/ERK pathways

**Fig. 6:**
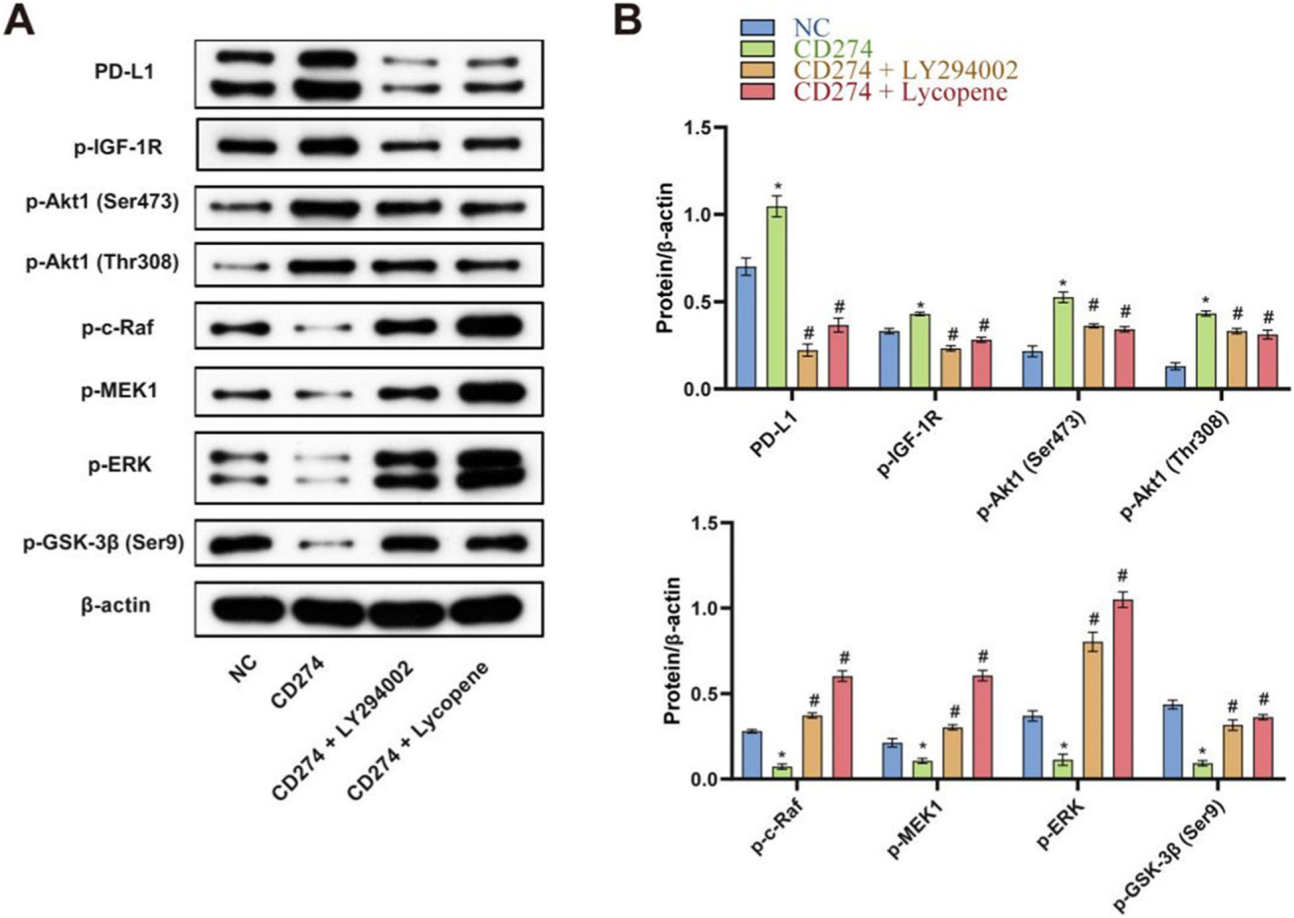
Effects of CD274 overexpression, LY294002, and lycopene on PI3K/AKT and Raf/MEK/ERK pathways. **A** Western bolt was performed to analyze PD-L1 and downstream key molecules p-IGF-1R, p-Akt1 (Ser473), p-Akt1 (Thr308), p-c-Raf, p-MEK1, p-ERK, and p-GSK-3β (Ser9) expressions. According to manufacturer’s instructions, there were two bands of PD-L1, p-ERK owing to post-translation modification. **B** Quantitative statistical analysis of **A**. *P < 0.05 vs NC, # *P* < 0.05 vs CD274. NC means negative control

Previous studies reported that in some cells, PTEN mutation might contribute to the suppression of the Raf/MEK/ERK cascade due to the capability of elevated activated Akt to phosphorylate and inactivate Raf1 [17]. Therefore, we tested this mechanism in TSCC CAL27 cells by using PI3K-Akt inhibitor, LY294002. The results showed that PD-L1 protein expression increased after overexpression of PD-L1, then p-IGF-1R elevated, increasing p-Akt1 (Ser473) and p-Akt1 (Thr308), indicating activation of the PI3K/AKT pathway, but expression of p-c-Raf, p-MEK1, and p-ERK decreased. The effect on the critical EMT controlling molecule p-GSK-3β was a most dramatic decrease. After adding PI3K-Akt inhibitor, LY294002, PD-L1 protein expression decreased, then p-IGF-1R decreased, decreasing p-Akt1 (Ser473) and p-Akt1 (Thr308), indicating inactivation of the PI3K/AKT pathway; interestingly, the expression of p-c-Raf, p-MEK1, and p-ERK increased as expected; these observations confirmed the activated PI3K/AKT pathway depressing the Raf/MEK/ERK cascade in CAL27 cells. The effect on the critical EMT controlling molecule p-GSK-3β was a most dramatic increase. The use of 4 μM lycopene after D-L1 overexpression produced a comprehensive effect similar to the LY294002 treatment (Fig. 6A and B). These results confirmed that PD-L1 upregulation activated Akt and depressed the Raf/MEK/ERK cascade in CAL27 cells.

## Discussion

Although researchers have made great efforts to explore the mechanisms behind PD-L1 expression and signal transduction in cancer, the definite mechanisms are still not fully elucidated, especially in TSCC. Understanding the mechanism of tumor-promoting PD-L1 expression and signal transduction in TSCC may provide therapeutic opportunities for improving PD-L1 overexpression-induced intratumor immunosuppression and overcoming resistance to PD-1/PD-L1 antibody-targeted therapy [6]. This study assumes the responsibility of supplying materials for identifying and reversing tumor-intrinsic constitutive mechanisms responsible for controlling PD-L1 expression and promoting tumor progression.

There are several intrinsic and extrinsic mechanisms responsible for PD-L1 regulation in tumor cells, including genetic alterations such as disruption of the 3’ untranslated region [19], copy number gains and deletions [20], epigenetic modifications such as microRNAs [21], promoter DNA methylation [22], and histone modifications; inflammatory cytokines and other factors such as IFN-γ, TNF-α, and TGF-β; hypoxia in the tumor microenvironment; and most importantly oncogenic and tumor suppressor signals [6]. PI3K/ATK/mTOR and Ras/MAPK pathway activation are involved with constitutive PD-L1 regulation in many cancers; loss of PTEN [23] or mutations in PIK3CA [24] lead to PI3K/ATK/mTOR pathway activation, while mutations in EGFR or Ras [25, 26] lead to Ras/MAPK pathway activation. Moreover, oncogenic transcription factors including NF-κB, MYC, STAT, IRF-1 [27], AP-1 [28], and HIF [29] have been demonstrated to modulate PD-L1 expression at the transcriptional level [6]. Intriguingly, lycopene might inhibit the carcinogenic process via the inactivation of the growth factor (IGF, VEGF, and PDGF)-induced PI3K/AKT/mTOR and Ras/RAF/MAPK signaling pathways [16]. What is more, lycopene could further prevent the Ras/MAPK and PI3K/AKT pathways from transducing signals into the nucleus to activate the aforesaid oncogenic transcription factors [16]. We achieved exogenous PD-L1 overexpression in CAL27 cells to find PD-L1 protein expression increased. P-IGF-1R increased significantly after PD-L1 overexpression, which confirmed involvement of the IGF system; its activation could activate the PI3K/AKT/mTOR and Ras/Raf/MAPK cascades and be downregulated by lycopene.

Previous studies reported that in some cells, PTEN mutation might contribute to suppression of the Raf/MEK/ERK cascade due to the capability of elevated activated Akt to phosphorylate and inactivate Raf1 [17]. We had tested this mechanism by using PI3K-Akt inhibitor LY294002 and found that p-c-Raf, p-MEK1, and p-ERK expressions elevated, which confirmed PD-L1 upregulation activated Akt and depressed the Raf/MEK/ERK cascade in CAL27 cells. Our study on SCC9 cells found that PD-L1 knockdown simultaneously downregulated the PI3K-Akt and Raf/MEK/ERK cascades; the lycopene treatment produced a similar effect, and the combination treatment aggravated the effect of PD-L1 knockdown. Study on glioblastoma multiforme found that PD-L1 prominently activated EMT process via binding and activating Ras in a MEK/ERK-dependent but PI3K/Akt-independent manner [14]. These results suggest three ways of PD-L1 upstream signaling that are PI3K-activated Akt pathway, Ras-activated Raf/MEK/ERK pathway, or simultaneous activation of both pathways, which might require different treatments, but all converge to activate NF-κBp65 to promote EMT.

FOXOs (forkhead box proteins, class O subgroup) are generally considered to be tumor suppressors, which are essential for cancer initiation and progression. FOXO1 silencing by siRNA in hepatocellular carcinoma reduced epithelial and enhanced mesenchymal marker expression indicating EMT. The same study also revealed that EMT induced by ZEB-2 could be suppressed by FOXO1 overexpression [30]. FOXO3 has been demonstrated to promote cell invasion and migration by inducing the expression of matrix metalloproteases (MMPs), including MMP-2, MMP-3, and MMP-9 in many studies [31]. FOXO1 and FOXO3 were confirmed to negatively regulate a set of angiogenesis-related and vascular remodeling genes, including angiopoietin 2 and eNOS [32]. In our study, PD-L1 overexpression or IGF-1R signaling induced p-Akt-mediated increase of p-FOXO1a and p-FOXO3a leading to their nuclear exclusion and degradation; consistently, the total FOXO1a and FOXO3a decreased. It is intriguing that in our study, activated p-Akt reduced P-GSK3β (Ser9) but increased p-FOXOs, which is different from the case in hepatocellular carcinoma where p-Akt increased P-GSK3β (Ser9) and p-FOXOs; concomitantly, this difference makes that active GSK3β in our study can not upregulate IGF-1R expression by FOXO1a and FOXO3a binding to IGF-1R promoter [33]. Phosphorylated FOXO1a and FOXO3a could be rescued by protein phosphatase 2A-mediated dephosphorylation and return to nucleus. Conversely, 4 μM lycopene decreased p-FOXO1A and p-FOXO3A and at the same time increase the level of FOXO1A and FOXO3A which might increase target gene activity to induce cell cycle arrest and apoptosis, to repair DNA damage and to suppress metastasis and angiogenesis. Combining 4-μM lycopene and PD-L1 overexpression attenuated the effect of PD-L1 signaling on FOXO1 and FOXO3. PD-L1 knockdown dramatically reduced p-FOXO1A and p-FOXO3A, which was further dramatically reduced by combination with 4 μM lycopene, which also meant much more FOXO1A and FOXO3A with tumor-suppressing activities.

GSK-3β is a serine/threonine kinase involved in various critical signaling pathways controlling cell survival, proliferation, and immunity, while also a crucial negative regulator of EMT [11]. Growth factors, such as PDGF, EGF and regulatory kinases in the upstream such as PI3K, AKT, and p70 ribosomal S6 kinase were reported to phosphorylate and inactivate GSK-3β [34]. Protein phosphatases such as PP2A and PP1 were reported to regulate GSK-3β activity by dephosphorylating the Ser9 phosphate group. In our study, exogenous PD-L1 overexpression upregulated IGF-1R and activated the PI3K/AKT pathway, due to the p-AKT inhibitory effect, the Raf/MEK/ERK pathway was inactivated; the total effect was p-GSK-3β (Ser9) dramatically decreased to a very low level, suggesting a negative control which in turn dramatically elevated NF-κBp65, STAT3, c-Myc, Snail, Slug, and ZEB-1 activity. Lycopene (4 μM) significantly increased p-GSK-3β (Ser9); the combination of PD-L1 overexpression and 4 μM lycopene neutralized the increasing and decreasing effects to produce a moderate decrease of p-GSK-3β (Ser9). PD-L1 knockdown more significantly upregulated p-GSK-3β (Ser9) than 4 μM lycopene, which was further elevated by combination with lycopene. These detailed data verified that GSK-3β was indeed a crucial downstream molecule of PD-L1 constitutive signaling and function as key negative regulator of EMT.

It has been shown that PD-L1 overexpression reduced p-GSK-3β (Ser9), which meant more active GSK-3β because the total amount of GSK-3β was usually stable. Moreover, active GSK-3β was reported to phosphorylate and stabilize NF-κB essential modifier (NEMO), facilitating its interaction with IκB kinase (IκK). Activated IκK phosphorylated the NF-κB inhibiting IκBs, promoting their degradation via the proteasome pathway, and thus promoting the free NF-κB translocation to the nucleus where it promoted gene transcription [35]. Lycopene has been suggested to induce NF-κB inactivation via inhibiting the phosphorylation of IκKα and IκBα and via decreasing translocation of the NF-κBp65 subunit from the cytoplasm to the nucleus [16]. In our study, PD-L1 overexpression increased active GSK-3β to upregulate NF-κB and enable its translocation from cytoplasm to the nucleus. We can confirm NF-κB, FOXO1a, and FOXO3a subcellular localization directly by IHC and westblot in further studies; for now, it is an apparent limitation of the present study. MUC1-C induces ZEB-1 expression by occupying the ZEB-1 promoter with NF-κBp65 [36]. Moreover, MUC1-C binds directly to ZEB-1 and promotes ZEB-1 occupancy on target gene promoters [37]. Transcript factors NF-κBp65 and STAT3 and STAT3 and c-Myc have been reported to be interconnected [38, 39], and all might occupy the promoter of PD-L1 to promote its expression [40, 41]; in our study, PD-L1 overexpression upregulated and lycopene downregulated them concomitantly to mediate activating and inhibitory signaling. Our research also demonstrated that EMT transcript factors Snail, Slug, and ZEB-1 and markers E-cadherin, N-cadherin, and Vimentin were bidirectionally and precisely regulated by upstream signals mediated by the critical negative molecule p-GSK-3β.

Taken together, our study demonstrated that PD-L1 upregulation elevated IGF-1R to activate the PI3K/AKT pathway but inactivated the Raf/MEK/ERK pathway in CAL27 cells, while PD-L1 knockdown decreased IGF-1R to inactivate both PI3K/AKT and Raf/MEK/ERK pathways in SSC9 cells, then to increase/decrease p-FOXOs and decrease/increase p-GSK-3β, thus producing further changes in EMT, proliferation, migration, invasion, and apoptosis. Lycopene reversed PD-L1 signaling and expression by mechanisms opposite to PD-L1 upregulation but similar to PD-L1 knockdown. Our study provides another explanation that anti-PD-1 therapy might synergize with lycopene to downregulate tumor-intrinsic PD-L1 signaling and expression, which suggests that further investigations into different pathways and immune or nonimmune effects are urgently needed.

## Conclusions

Our study demonstrated that PD-L1 expression and signaling was significantly upregulated in the TSCC tissues. Further study identified PD-L1 signaling by different mechanisms to produce further changes in EMT, proliferation, migration, invasion, and apoptosis, whereas lycopene reversed its signaling and expression by the same and different mechanisms to produce the opposite effects in tongue squamous cell line CAL27 and SCC-9 cells. We explained the definite mechanisms underlying the previous reported phenomenon that lycopene dose-dependently suppressed the growth of CAL27 xenograft tumors [8]. These results provide a, to some extent, sounder basis for comprehending PD-L1 signaling and expression as well as prevention and treatment of TSCC.

## Abbreviations

AKT: Thymoma viral proto-oncogene
AP-1: Activator protein-1
EMT: Epithelial–mesenchymal transition
EMT-TFs: EMT-promoting transcription factors
eNOS: Endothelial nitric oxide synthase
ERK: Extracellular signal-regulated kinase pathway
FOXOs: Forkhead transcription factors, class O subgroup
GSK-3: Glycogen synthase kinase-3β
HIF: Hypoxia-inducible factor
IFN-γ: Interferon-γ
IGF-I: Insulin-like growth factor-I
IGF-IR: Insulin-like growth factor-I receptor
IL-6: Interleukin-6
IRF-1: Interferon regulatory factors 1
MAPK/MEK: Mitogen-activated protein kinase kinase/mitogen extracellular kinase
MET: Mesenchymal–epithelial transition
MTORC1: Mechanistic target of rapamycin kinase complex 1
MUC1: Mucin antigen 1
NF-kB: Nuclear transcription factor-kappa B
PDGF: Platelet-derived growth factor
PD-L1: Programmed death-ligand 1
PD-1: Programmed death receptor 1
PI3K: Phosphoinositide 3-kinase
PTEN: Phosphatase and tensin homolog deleted on chromosome 10
RAF: RAF proto-oncogene
RAS: Ras proto-oncogene
STAT3: Signal transducer and activator of transcription 3
TGF-β: Transforming growth factor-β
TNF-α: Tumor necrosis factor α
TSCC: Tongue squamous cell carcinoma
TUNEL: Transferase-mediated dUTP nick end labeling assay
VEGF: Vascular endothelial growth factor
